# Cretaceous origin of dogwoods: an anatomically preserved *Cornus* (Cornaceae) fruit from the Campanian of Vancouver Island

**DOI:** 10.7717/peerj.2808

**Published:** 2016-12-21

**Authors:** Brian A. Atkinson, Ruth A. Stockey, Gar W. Rothwell

**Affiliations:** 1 Department of Botany and Plant Pathology, Oregon State University, Corvallis, OR, United States; 2 Department of Environmental and Plant Biology, Ohio University, Athens, OH, United States

**Keywords:** Campanian, Cornales, Cornelian cherries, Dogwoods, *Cornus*, Fruit

## Abstract

**Background:**

Cornaceae consists of 58 species, all within the genus *Cornus*. The Cenozoic record of *Cornus* is extensive and well documented. Molecular divergence-time studies suggest that crown-group *Cornus* may have originated by the Late Cretaceous. However, there has been no formal report of *Cornus* from Cretaceous deposits. Here, we characterize a permineralized fossil fruit assignable to *Cornus* subg. *Cornus* from the Upper Cretaceous (Campanian) Shelter Point locality of Vancouver Island, British Columbia, Canada.

**Methods:**

Serial sections of the specimen were made using the cellulose acetate peel technique. Peels were mounted onto microscope slides and studied by light microscopy.

**Results:**

The fossil fruit consists of a tri-locular woody endocarp with dorsal germination valves. The locules are sub-triangular to ellipsoidal in transverse section and are separated by thin septa. Endocarp tissue consists of elongated and isodiametric sclereids and secretory cavities. Internal vascular tissue was not observed, but is interpreted to have been located along the outer periphery of the septa for some length, common in many cornalean taxa. There is one seed in each locule, one of which was found to have endosperm and a dicotyledonous embryo.

**Discussion:**

Woody endocarps with germination valves, without central vascular bundles, and with one seed per locule are characteristic of several families within the order Cornales. The interpreted vascular pattern and presence of secretory cavities indicates that the fossil fruit is assignable to *Cornus* subg. *Cornus*. Comparative analysis suggests that the fossil is most similar to *Cornus piggae*, a species described from the Paleocene of North Dakota. This fossil is the first evidence of crown-group Cornaceae from the Cretaceous and sheds light on both the plesiomorphic fruit characters and the timing of the initial diversification of the family and basal asterid lineage, Cornales.

## Introduction

The family Cornaceae comprises 58 species of trees, shrubs, and rhizomatous herbs, commonly known as dogwoods, within the genus *Cornus* L. (Eyde, 1987; Eyde, 1988; Xiang et al., 1993; Xiang et al., 2006; Murrell, 1993; Xiang, Soltis & Soltis, 1998). Dogwoods are broadly distributed across Eurasia, North America, northern South America, and sub-Saharan eastern Africa (Eyde, 1988; Murrell, 1993; Murrell, 1996; Xiang, Shui & Murrell, 2003). There are four major clades within *Cornus* supported by both morphological and molecular characters: the blue- or white- fruited dogwoods, big-bracted dogwoods, dwarf dogwoods, and cornelian cherries (Eyde, 1987; Eyde, 1988; Xiang et al., 2006). Each of these groups can be distinguished based on fruit morphology and anatomy (see Eyde, 1988).

Throughout the past decade, molecular phylogenetic studies have made significant progress towards our understanding of the evolutionary patterns and relationships of *Cornus* (Xiang et al., 1993; Xiang et al., 1996; Xiang et al., 2002; Xiang et al., 2005; Xiang et al., 2006; Xiang et al., 2008; Xiang, Soltis & Soltis, 1998; Xiang & Thomas, 2008; Feng, Xiang & Franks, 2011). Molecular divergence-time analyses, that used Cenozoic fossil calibrations, suggested a Late Cretaceous origin for Cornaceae, and that the four major clades of *Cornus* diverged and diversified by the latest Cretaceous or early Paleogene (Xiang et al., 2005; Xiang et al., 2006; Xiang, Thomas & Xiang, 2011). However, there have been no *Cornus* fossils formally described from Cretaceous deposits to date (see Manchester et al., 2009).

*Cornus* has an extensive Cenozoic record (Eyde, 1988; Manchester, Xiang & Xiang, 2010). The most widely accepted, earliest described fossils of the genus consist of leaves of *Cornus swingii* Manchester et al. (2009) and *C. krassilovii* Manchester et al. (2009) from the Paleocene of North America and Asia, respectively, and fruits described as *Cornus piggae* Manchester, Xiang & Xiang (2010) from the Paleocene of North Dakota, USA. In addition, Manchester & Kapgate (2014) recently reported a fruit resembling a cornelian cherry (*Cornus* subg. *Cornus*) from the K/Pg boundary of India, which is currently being studied (SR Manchester, pers. comm., 2016). Given that fruit characters of *Cornus* are systematically informative (Eyde, 1988; Xiang, Shui & Murrell, 2003; Manchester, Xiang & Xiang, 2010; Morozowska, Gawrońska & Woźnicka, 2013; Woźnicka, Melosik & Morozowska, 2015), fossil fruits of this genus have great potential for revealing ancient evolutionary patterns and relationships.

As part of a broader initiative to understand the earliest evolutionary patterns and relationships of the order Cornales (see Atkinson, 2016; Stockey, Nishida & Atkinson, 2016), we describe a permineralized fruit assigned to *Cornus* cf. *piggae* from the Cretaceous (Campanian ∼73 Ma) of Vancouver Island. This fruit is the oldest known dogwood fossil to date and provides a new minimum age for the diversification of crown-group *Cornus*. The presence of an Upper Cretaceous *Cornus* has important implications for our understanding of both the plesiomorphic characters of Cornaceae and the early diversification of Cornales.

## Materials and Methods

A single permineralized fruit was recovered from the Shelter Point locality on Vancouver Island, British Columbia, Canada. The exposure at Shelter Point consists of six units, two of which contain permineralized fossil plants (Richards, 1975). Sediments are part of the Upper Cretaceous Spray Formation of the Nanaimo Group, which is considered upper Campanian based on the presence of *Longusorbis* decapod fossils (Richards, 1975). Plant material at Shelter Point is rare, and preserved in calcareous concretions. Pinaceous seed cones of *Pityostrobus beardii* Smith & Stockey (2002) and cyatheaceous tree fern remains of *Rickwoodopteris hirsuta* Stockey & Rothwell (2004) have been described from this locality.

The concretion containing the study specimen was cut into slabs using a water-cooled saw with a diamond-edged blade. The fossil fruit was exposed on one face of a slab, and was subsequently sectioned using the cellulose acetate peel technique (Joy, Willis & Lacey, 1956). Peels were mounted on microscope slides using Eukitt (O. Kindler GmbH, Freiberg, Germany) xylene soluble mounting medium. Photographs were taken with a digital Better Light (Placerville, CA) scanning camera mounted on a Leitz Aristophot large format camera, and focused through either Summar lenses or a Zeiss WL compound microscope. Images were processed with Adobe Photoshop CS 5.0 (Adobe, San Jose, California, USA). Microscope slides are housed in the paleontology collections of the Royal British Columbia Museum, Victoria, British Columbia, Canada.

### Systematic paleobotany

**Table:** 

Order—Cornales
Family—Cornaceae (sensu Xiang et al., 2002)
Genus—*Cornus* L.
Subgenus—*Cornus* (sensu Xiang et al., 2005; Xiang et al., 2006)
Species—*Cornus* cf. *piggae*Manchester, Xiang & Xiang (2010)

*Repository.* Royal British Columbia Museum, Victoria, British Columbia, Canada.

*Locality.* Beach at Shelter Point, Vancouver Island, British Columbia, Canada (49°56^′^39^′′^N, 125°11^′^10^′′^W).

*Stratigraphic Position and Age.* Spray Formation, Late Campanian (∼73 Ma).

#### Description

**Figure 1:**
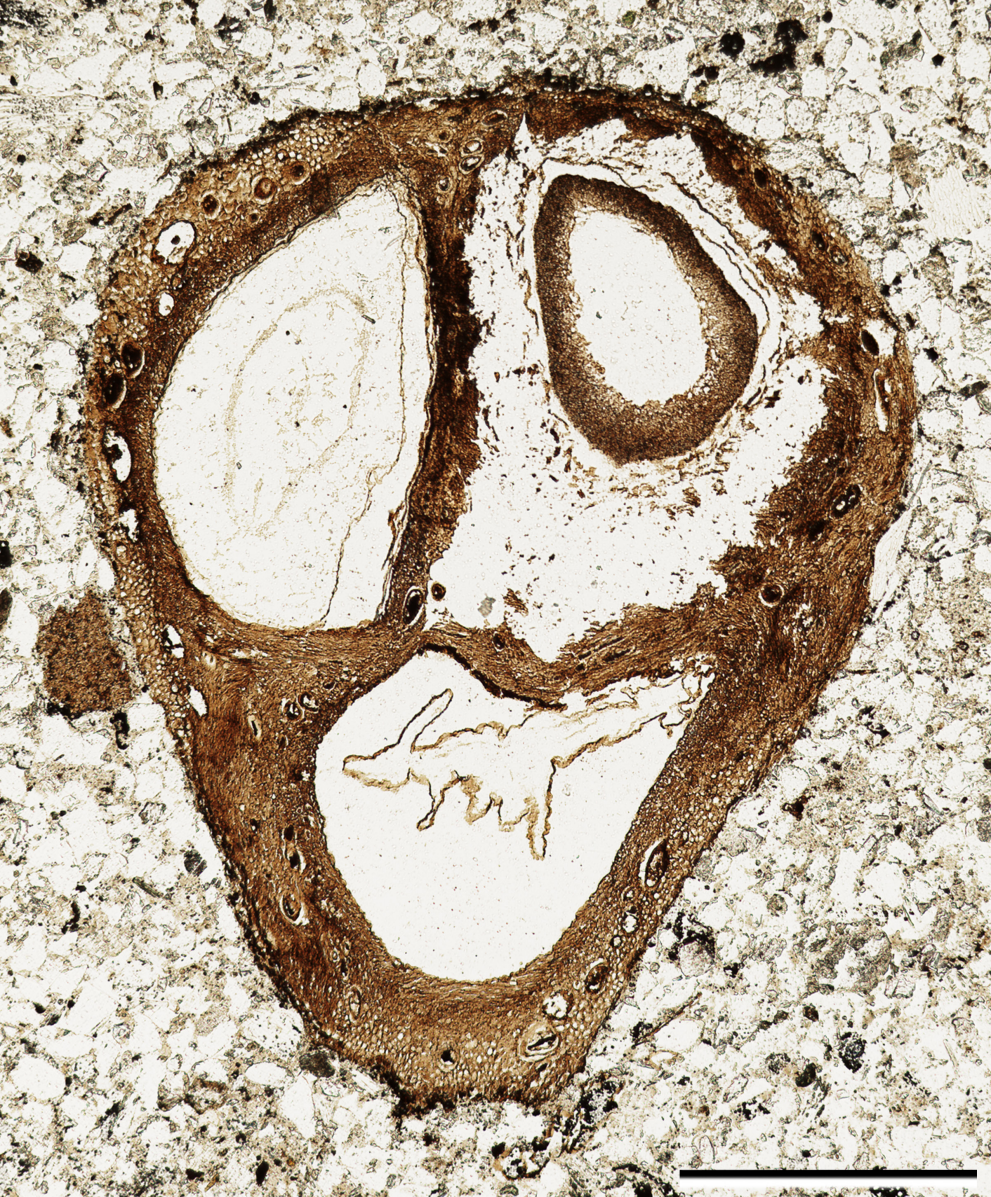
General fruit morphology. Cross section of fruit showing endocarp with three locules and one seed per locule. Note numerous secretory cavities within endocarp tissue. SH 790 B1 Bot #10. Scale = 1.0 mm.

**Figure 2:**
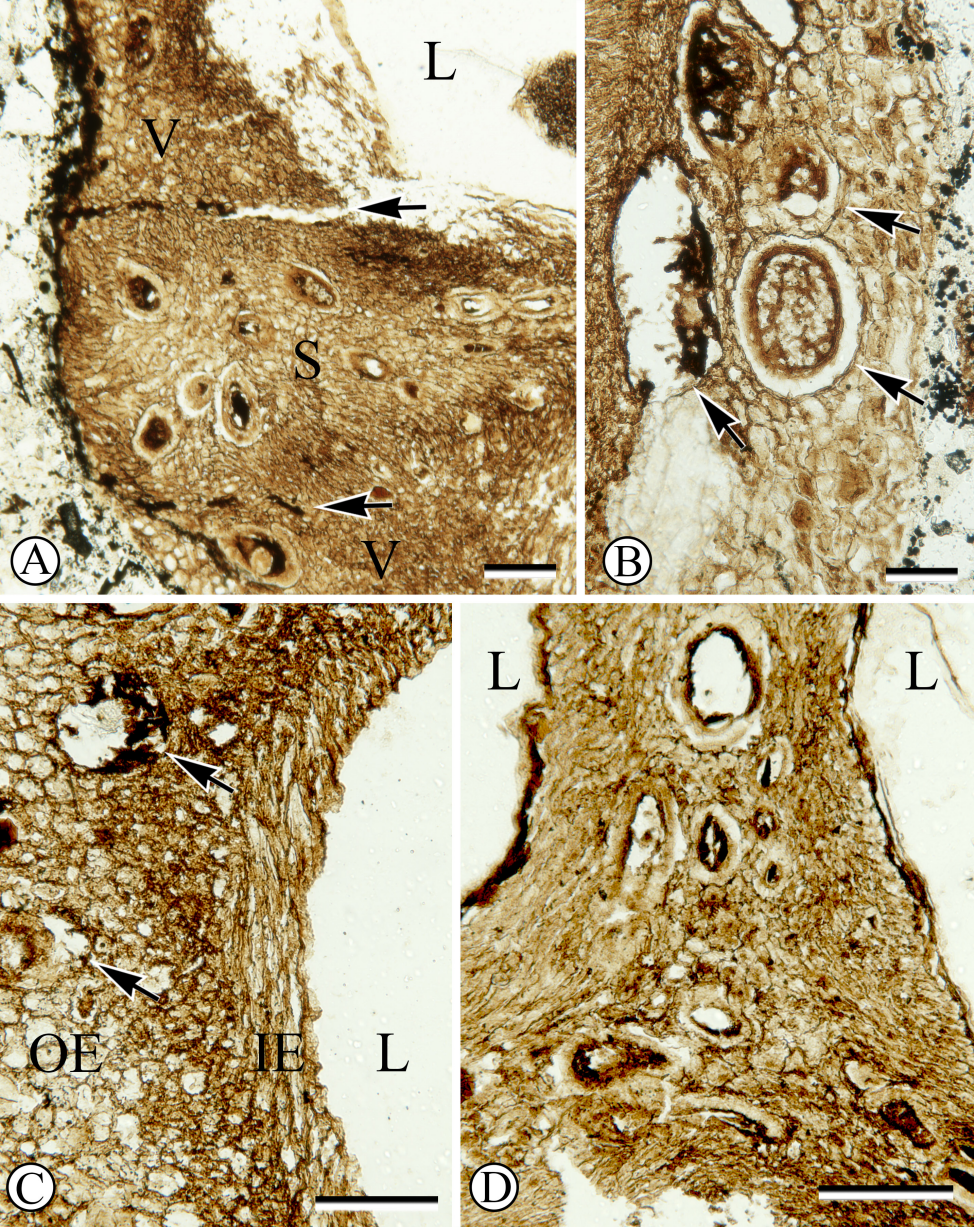
Anatomy of endocarp tissues. (A) Cross section of endocarp wall towards base showing planes of weakness of two germination valves (V) separated by septum (S), and locule (L). SH 790 B1 Bot #34. Scale = 100 µm. (B) Cross section of valve showing isodiametric sclereids and secretory cavities (arrows). SH 790 B1 Bot #34. Scale = 60 µm. (C) Cross section of valve showing inner endocarp (IE) with elongated sclereids tangential to locule (L), outer endocarp (OE) with isodiametric sclereids and secretory cavities (arrows). SH 790 B1 Bot #4. Scale = 50 µm. (D) Cross section of endocarp showing locules (L) and central axis with inner endocarp tissue (elongated sclereids tangential to locules) and outer-endocarp tissue (secretory cavities and isodiametric sclereids). Note absence of central vascular bundle. SH 790 B1 Bot #33. Scale = 230 µm.

The fossil fruit consists of a tri-locular woody endocarp with preserved seeds (Fig. 1). One of the locules contains a fungal structure (Fig. 1), and hyphae can be seen in several places within the locule. Although the apex of the fruit was lost in the saw cut, the remaining endocarp is 1.3 mm long and 4.0 mm wide. The exterior surface of the endocarp is smooth, without conspicuous grooves or ridges. Locules are ellipsoidal to sub-triangular in cross section (Fig. 1) and at least 2.0 mm in diameter. Each locule has a dorsal germination valve, 0.4–0.5 mm thick, that extends the length of the fruit (Fig. 2A). Septa are relatively thin, 0.2–0.5 mm thick (Figs. 1, 2A, 2D).

The ground tissue of the endocarp consists of sclerenchyma in the form of isodiametric and elongated sclereids (Figs. 2A–2D). The elongated sclereids, 120 µm long and 12–24 µm wide, are often circum-locular (sometimes longitudinally elongated), and form a distinct multiseriate layer (Figs. 1, 2B–2D). This layer is interpreted as the inner endocarp (sensu Morozowska, Gawrońska & Woźnicka, 2013; Morozowska & Wysakowska, 2016). Outside of the inner endocarp is a zone of isodiametric sclereids, 18–30 µm wide, and secretory cavities, 50–100 µm wide, that form a uniseriate cycle around each locule (Figs. 1, 2A–2D). This tissue is designated as the outer endocarp (sensu Morozowska, Gawrońska & Woźnicka, 2013; Morozowska & Wysakowska, 2016).

There is no central vascular bundle or any other internal vascular tissue that can be identified within the endocarp. Many taxa within Cornales, including *Cornus*, have endocarps with no internal vasculature for much of their length; however, bundles run in the mesocarp along the outer periphery of the septa, for some distance before entering the endocarp towards the apex and traversing the septa to supply the seeds (Horne, 1914; Wilkinson, 1944; Eyde, 1963; Eyde, 1967; Eyde, 1988; Manchester, Xiang & Xiang, 2010; Woźnicka, Melosik & Morozowska, 2015; Atkinson, 2016; Stockey, Nishida & Atkinson, 2016). Although the apex of the fossil fruit is missing, due to the conspicuous absence of any internal vascular tissue, it is most likely that this endocarp had a similar vascular pattern.

**Figure 3:**
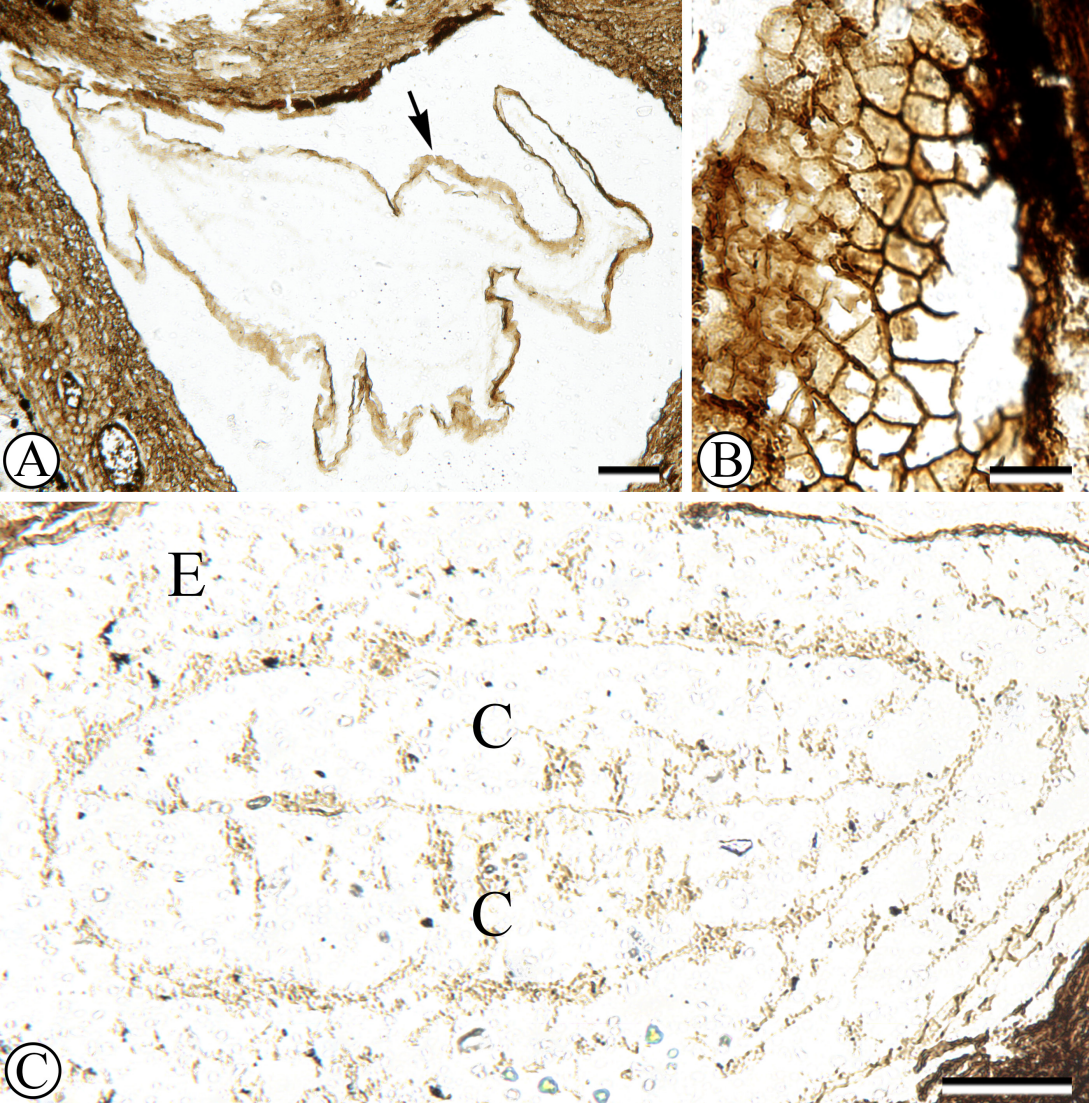
Seed structure. (A) Oblique cross section of seed with membranous seed coat (arrow). Sh 790 B1 Bot #15. (B) Peridermal section of integument. SH 790 B1 Bot #1. (C) Cross section of seed with endosperm (e) surrounding embryo with two cotyledons (c). Sh. 790 B1 Bot #25.

Each locule has one seed enclosed with a membranous seed coat that is one cell layer thick (Figs. 1, 3A). Integumentary cells are short and irregularly shaped (cuboidal to polygonal) in paradermal section (Fig. 3B). One seed in the fruit has a dicotyledonous embryo that is surrounded by remains of endosperm (Figs. 1, 3C). Cotyledons are spathulate, and measure 900 µm wide by 180 µm thick in transverse section.

One seed is heavily colonized by fungi (Fig. 1, at upper right). Endosperm and embryo tissues are not preserved in this seed. The fungal structure has a hollow center; towards the outside the fungal hyphae form pseudoparenchyma, and towards the inside cellular patterning becomes disorganized.

## Discussion

The fossil fruit from the Campanian Shelter Point locality on Vancouver Island consists of a sclerenchymatous endocarp with dorsal germination valves, without a central vascular bundle, and one seed per locule. This suite of fruit characters is characteristic of several taxa within Cornales (Eyde, 1963; Eyde, 1967; Eyde, 1988; Takahashi, Crane & Manchester, 2002; Manchester, Xiang & Xiang, 2010; Xiang, Thomas & Xiang, 2011; Atkinson, 2016; Stockey, Nishida & Atkinson, 2016). More specifically, the endocarp of the Shelter Point fruit contains isodiametric and elongated sclereids, as well as secretory cavities, which are characteristic of some species within Cornaceae (Eyde, 1988; Takahashi, Crane & Manchester, 2002; Manchester, Xiang & Xiang, 2010; Atkinson, 2016; Stockey, Nishida & Atkinson, 2016), and the extinct taxon *Suciacarpa starrii* Atkinson (2016).

Living species of Cornaceae belong to four major clades within the genus *Cornus* L.: cornelian cherries, big-bracted dogwoods, dwarf dogwoods, and blue- or white-fruited dogwoods, (Eyde, 1988; Xiang et al., 2006). All four of these clades have endocarps with isodiametric sclereids, ellipsoidal to sub-triangular locules in cross section, and vascular bundles located at the periphery of the septa (Table 1; Wilkinson, 1944; Eyde, 1967; Eyde, 1988). The cornelian cherry clade, *Cornus* subg. *Cornus* (sensu Xiang et al., 2005),  is the only lineage, however, with endocarps that contain both secretory cavities and elongated sclereids (Table 1; Eyde, 1987; Eyde, 1988; Xiang, Shui & Murrell, 2003; Manchester, Xiang & Xiang, 2010). These characters are shared by the Shelter Point fruit, and confirm its affinity with the cornelian cherry clade.

**Table 1:** General endocarp comparisons of dogwood fruits and close relatives.

	Ridges and/or grooves on valve surface	Locule shape in x.s.	Elongate sclereids	Secretory cavities	Vasculature
***Cornus* cf.* piggae***^a^	**Absent**	**Ellipsoidal/subtriangular**	**Present**	**Present**	**Periphery of septum?**
Blue/White-fruited dogwoods	Present	**Ellipsoidal/subtriangular**	Absent	Absent	**Periphery of septum**
Big-bracted dogwoods	Present	**Ellipsoidal/subtriangular**	Absent	Absent	**Periphery of septum**
Dwarf dogwoods	**Absent**	**Ellipsoidal/subtriangular**	Absent	Absent	**Periphery of septum**
Cornelian cherries	Present/**absent**	**Ellipsoidal/subtriangular**	**Present**	**Present**	**Periphery of septum**
*Suciacarpa starrii*	**Absent**	Crescent	**Present**	**Present**	Rows of bundles within septum

**Notes.**

Data from: Wilkinson (1944), Eyde (1967), Eyde (1988), Manchester, Xiang & Xiang (2010) and Atkinson (2016).

a Specimen described in this paper.

There are six extant species of cornelian cherries: *C. chinensis* Wangerin, *C. eydeana* Xiang & Shui, *C*. *mas* L., *C*. *officianalis* Sieb. et Zucc., *C. sessilis* Torr. ex Durand, *C*. *volkensii* Harms (Eyde, 1988; Xiang et al., 2005; Manchester, Xiang & Xiang, 2010). In addition, there are three previously described extinct species of cornelian cherries based on silicified and pyritized endocarps: *C*. *piggae* Manchester, Xiang & Xiang from the Paleocene of North America, and *C. ettingshausenii* (Gardner) Eyde and *C. multilocularis* Gardner (Eyde) from the Eocene London Clay localities of England (Reid & Chandler, 1933; Eyde, 1988; Manchester, Xiang & Xiang, 2010).

One of the more obvious differences between endocarps of extant and extinct species of *Cornus* subg. *Cornus* is the number of locules. Living species commonly have one to two locules per endocarp (rarely three); whereas extinct species, including the fossil described in this study, frequently have more than two locules per endocarp (Table 2; Eyde, 1988). Another character that has been used in comparing endocarps of cornelian cherries is the wall thickness percentage (Wtp in Table 2), which is the thickness of the endocarp wall (germination valve) divided by the diameter of the endocarp, multiplied by 100 (Manchester, Xiang & Xiang, 2010). It is worth noting that caution should be taken while using this character for comparative analysis of fossils because of the possibility of measuring heavily eroded endocarps, which could lead to inaccurate wall thickness perecentages. The fossil endocarp in this study has a more or less uniform wall thickness; thus, we do not suspect that there was significant abrasion. While observing wall thickness percentages across the cornelian cherry clade, it appears that the majority of endocarps have relatively high values (Table 2; Manchester, Xiang & Xiang, 2010), while *C. piggae, C. multilocularis*, and the Shelter Point fruit have low values (Table 2).

**Table 2:** Endocarp morphology of cornelian cherries, *Cornus* Subgenus *Cornus*.

Taxa	Age	Locule No.	Length (mm)	Width (mm)	Length/width ratio	Apical depression	Vascular bundle exposure	Germination valve (wall) thickness (mm)	Wtp^b^
***Cornus* cf.* piggae***^a^	**Campanian**	**3**	**1.3**+	**4**	**?**	**?**	**?**	**0.5**	**12.5**
*Cornus piggae*	Paleocene	2–**3**	5–10	**5**–7	1–1.6	Absent	Apical half	**0.5**	**13**
*Cornus ettingshausenii*	Eocene	**3**(−5)	14	13	1	Present	Apical half	2.3	27
*Cornus multilocularis*	Eocene	**3**–6	5–17	8–12.5	1.4–1.6	Present	Apical half	0.9	**12**
*Cornus chinensis*	Recent	2	7.5	**4**	1.8	Present	Basal half	**0.6**	23
*Cornus eydeana*	Recent	2	20–25	7–8	3	Present	Basal half	1.8	24
*Cornus mas*	Recent	2 (1–**3**)	9–20	**4**–7.3	2.2–2.7	Present	Apical half	1.2	20
*Cornus officianalis*	Recent	1	11–18	6–11.5	1.5–1.8	Present	Apical half	0.9	34
*Cornus sessilis*	Recent	2	11	**4.2**	2.6	Absent	Apical half	1.2	28
*Cornus volkensii*	Recent	2	8	**4**	2	Present	Basal half	0.9	21

**Notes.**

Modified from Manchester, Xiang & Xiang (2010).

a Specimen described in this paper.

b Wall (germination valve) thickness percentage (Wtp) is calculated by the thickness of the germination valve divided by the diameter of the endocarp × 100.

The endocarps of *C*. *multilocularis* can be distinguished from those of the Shelter Point fruit by endocarp size and locule numbers (Table 2). *Cornus multilocularis* has large endocarps with a diameter of at least 8.0 mm and a valve thickness of at least 0.9 mm, almost double that of the Shelter Point endocarp (Table 2). Furthermore, the septa of *C. multilocularis* are thicker than those of the Shelter Point fruit (see Manchester, Xiang & Xiang, 2010). The endocarps of *C. multilocularis* typically have four to six locules, but rarely three (Manchester, Xiang & Xiang, 2010) as in the Shelter Point endocarp.

The endocarp of the Shelter Point fruit is most similar to those of *C. piggae* from the Paleocene of North Dakota (Table 2). Similar to the Shelter Point fruit, endocarps of *C*. *piggae* often have three locules (two is less common) (Table 2). The germination valves of both the Shelter Point fruit and those of *C. piggae* are 0.5 mm thick (Table 2). Furthermore, the wall thickness percentages of the Shelter Point fruit and *C. piggae* are indistinguishable given the sample size (Table 2). Although the apex of the Shelter Point fruit is missing, the available data reveals striking similarities between the Shelter Point endocarp and those of *C. piggae.* The presence or absence of an apical depression in the endocarp, the length of the endocarp, and the amount of exposure of vascular bundles on the outer periphery of the septa are important taxonomic characters that may distinguish the Shelter Point fossil from *C. piggae*, but cannot be determined at this time (Table 2). Additional specimens are needed to confidently assign the Shelter Point fruit to either *Cornus piggae* or to a new species of *Cornus*. Although our assignment of the Shelter Point fossil to *Cornus* cf. *piggae* remains tentative, we nonetheless document that the *C. piggae*-type fruits are, so far, the most ancient of Cornaceae.

*Suciacarpa starrii* Atkinson (2016) from the Campanian of North America is the only other known cornalean outside of the cornelian cherry clade that has endocarps with secretory cavities (Table 1), and should be discussed briefly. Endocarps of *S. starrii* can be distinguished from those of *C*. *piggae* by several characters. While fruits of *Suciacarpa* have four crescent-shaped locules and rows of vascular bundles within the septa (Atkinson, 2016), those of *C. piggae* (including the fossil in this study) have three ellipsoidal to sub-triangular locules and lack rows of vascular bundles within the septa (Manchester, Xiang & Xiang, 2010). Thus, in number of locules, shape of locules, and position of vascular bundles, fruits of *Suciacarpa* differ from *C. piggae* and also from the Shelter Point fruit (Table 1). Atkinson (2016) speculated that *Suciacarpa* may represent an extinct member of Cornaceae, however its phylogenetic position within the order Cornales is uncertain at this time.

### Early evolutionary patterns of dogwoods and other cornaleans

*Cornus* cf. *piggae* from the late Campanian of western North America is the oldest known fossil of *Cornus* subg. *Cornus* and crown group Cornaceae to date. *Cornus piggae* was first described from silicified endocarps preserved in upper Paleocene deposits of central North America (Manchester, Xiang & Xiang, 2010) and was previously recognized as the oldest representative of the cornelian cherry clade. The occurrence of *C*. cf. *piggae* on Vancouver Island, Canada documents that the cornalean cherries originated well before the K/Pg boundary. The endocarp described in this study provides a minimum age for the subgenus *Cornus* of 73 Ma, and extends the fossil record of *Cornus* and Cornaceae by at least 12 million years. This minimum clade age for *Cornus* subg. *Cornus* is congruent with the latest divergence time estimate of 73.4 Ma for the split between the cornelian cherry clade and the big-bracted and dwarf dogwood clade (Xiang & Thomas, 2008).

Molecular divergence-time calculations predict that Cornaceae diverged from their sister group, Alangiaceae, around 80 Ma (Xiang & Thomas, 2008; Xiang, Thomas & Xiang, 2011) and that the family radiated into its four major clades by the end of the Cretaceous (Xiang & Thomas, 2008). The fossil cornelian cherry described in this study is the first fossil evidence for crown-group Cornaceae during the Cretaceous, providing empirical support for the clade age calculation of previous studies (Xiang & Thomas, 2008; Xiang, Thomas & Xiang, 2011) and rejecting the hypothesis of a Paleogene origin for the family and sub-clade (Xiang et al., 2006; Xiang & Thomas, 2008).

The geographic distribution of *Cornus* is characterized by several intercontinental disjunctions which have been the subject of a number of studies and biogeographic analyses (e.g., Eyde, 1988; Xiang et al., 1996; Xiang et al., 2000; Xiang et al., 2005; Xiang et al., 2006; Xiang & Thomas, 2008; Manchester et al., 2009; Manchester, Xiang & Xiang, 2010). Previous analyses have cautiously (see Xiang & Thomas, 2008) concluded that Europe was either the ancestral area or the site of initial diversification of *Cornus* (Xiang et al., 1996; Xiang et al., 2005; Xiang & Thomas, 2008) because of the presence of Cenozoic fossils representing each sub-group. The intercontinental disjunctions of extant *Cornus* are often thought to be products of migrations over high latitude land bridges during the Paleogene and long distance dispersal events from Europe. However, recent discoveries and descriptions of *Cornus* fossils offer a different perspective. The oldest fossils of *Cornus* subg. *Cornus*, are now known from the Campanian and Paleocene of North America (Manchester, Xiang & Xiang, 2010, and this study). In addition, Manchester & Kapgate (2014) reported a bi-locular cornelian cherry endocarp from the K/Pg boundary of India, demonstrating that the clade was geographically widespread early on in its evolutionary history. As of now, these ancient distributions indicate that Europe was not an ancestral area for cornelian cherries. *Cornus* subg. *Cornus,* and Cornaceae as a whole were probably more diverse and widely distributed in the past. These insights suggest that what remains of these clades may be ancient lineages with relictual distributions. We anticipate that as more fossils are recovered from Cretaceous and Paleocene deposits the paleogeographical distributions of cornaceous lineages will become more apparent, and that the evolutionary history of Cornaceae may prove to be more complex than previously thought.

The Late Cretaceous was a critical time for the initial phylogenetic radiation of the basal asterid order, Cornales (Knobloch & Mai, 1986; Mai, 1993; Takahashi, Crane & Manchester, 2002; Schenk & Hufford, 2010; Xiang, Thomas & Xiang, 2011; Manchester, Grímsson & Zetter, 2015; Atkinson, 2016; Stockey, Nishida & Atkinson, 2016). The most accepted earliest known cornalean fossils are fruits of *Hironoia fusiformis* Takahashi, Crane & Manchester (2002) from the early Coniacian of Japan. Recently, additional anatomically preserved cornalean fruits were recovered from Upper Cretaceous deposits including those of *Eydeia hokkaidoensis* Stockey, Nishida & Atkinson (2016) from the Santonian of Japan and the aforementioned *Suciacarpa starrii* (Atkinson, 2016) from the Campanian of North America. The phylogenetic relationships of these Coniacian, Santonian, and Campanian taxa are uncertain and at this point it appears that they probably represent stem lineages within Cornales (Atkinson, 2016; Stockey, Nishida & Atkinson, 2016).

Until recently the only reported evidence of crown cornalean lineages in the Cretaceous were fruits of Mastixiaceae from the Maastrichtian of Europe (Knobloch & Mai, 1986; Mai, 1993). However, in the past few years paleobotanical studies, including this one, have documented more cornalean fossil fruits from the upper Campanian of North America that represent crown-group families and genera. These include fossil fruits of *Cornus* (Cornaceae), *Davidia* Baill. (Davidiaceae), and Nyssaceae (Manchester, Grímsson & Zetter, 2015). This enhanced fossil record of Cornales documents that the primary diversification of extant cornalean families did indeed occur before the end of the Campanian, emphasizing the antiquity of some taxa.

## Conclusions

The current study is part of an ongoing series of investigations seeking to elucidate the early evolutionary patterns and relationships of the early diverging asterid order, Cornales (Atkinson, 2016; Stockey, Nishida & Atkinson, 2016). Each new fossil provides an empirical test for clade age and biogeographic hypotheses that are based on molecular trees of living species. The Campanian fruit described in this study is assigned to *Cornus* subg. *Cornus* cf. *piggae* and represents the oldest occurrence of Cornaceae to date and provides a minimum age of 73 Ma for the origin of subgenus *Cornus*. The discovery of this Cretaceous fossil and concurrent paleontological data suggest that the early biogeographic history of cornelian cherries as well as Cornaceae was probably more complex than previously realized, and that the ancestral area for the family remains uncertain.

## Additional Information and Declarations

### Data Availability

The following information was supplied regarding data availability: The research in this article did not generate, collect or analyse any raw quantitative data or code.
